# Identification of a novel germline *APC* N-terminal pathogenic variant associated with attenuated familial adenomatous polyposis

**DOI:** 10.1016/j.gendis.2023.101078

**Published:** 2023-09-07

**Authors:** Giovanna Forte, Valentina Grossi, Filomena Cariola, Antonia Lucia Buonadonna, Paola Sanese, Katia De Marco, Candida Fasano, Martina Lepore Signorile, Vittoria Disciglio, Cristiano Simone

**Affiliations:** a Medical Genetics, National Institute of Gastroenterology IRCCS “S. de Bellis” Research Hospital, Castellana Grotte, Bari 70013, Italy; b Medical Genetics, Department of Precision and Regenerative Medicine and Jonic Area (DiMePRe-J), University of Bari Aldo Moro, Bari 70124, Italy

Adenomatous polyposis coli (*APC*) is a key tumor suppressor gene playing a central role in the Wnt signaling pathway through β-catenin down-regulation.^1^ *APC* germline pathogenic variants are associated with familial adenomatous polyposis (FAP), an autosomal dominant colorectal cancer (CRC) predisposition syndrome characterized by the development of hundreds to thousands of colorectal adenomatous polyps.^1^ Depending on the number of polyps and age of onset, FAP can be classified into four forms: profuse FAP (>1000 adenomas), intermediate FAP (100-1000 adenomas), attenuated FAP (10–100 adenomas), and gastric polyposis and desmoid FAP (<50 polyps).^1^ Disease severity seems to be correlated with the location of *APC* pathogenic variants, most of which occur over the 5′ half of the gene, leading to the elimination of domains involved in β-catenin level regulation and Axin binding.^1^

Here, we report a 59-year-old woman with a novel pathogenic *APC* germline variant, who presented with an attenuated colonic polyposis phenotype and CRC. In an effort to decipher the correlation between molecular mechanisms and clinical phenotype, we clinically and molecularly characterized this novel *APC* N-terminal pathogenic variant and compared the index patient's phenotype with that of patients described in the literature carrying germline APC N-terminal truncating variants (codons 1–157).

In the current study, we examined a 59-year-old woman (Fig. 1A II:3) exhibiting an attenuated colonic polyposis phenotype and CRC, which was referred to our institution for genetic counseling. The patient had no family history of colorectal polyposis, CRC, or FAP-associated malignancies. Because of her medical history, her genomic DNA was extracted and subjected to genetic testing by next-generation sequencing using a panel of 25 hereditary cancer-related genes (*APC*, *ATM*, *BARD1*, *BMPR1A*, *BRIP1*, *CDH1*, *CDK4*, *CDKN2A*, *CHEK2*, *EPCAM*, *MLH1*, *MRE11A*, *MSH2*, *MSH6*, *MUTYH*, *NBN*, *PALB2*, *PMS2*, *PTEN*, *RAD50*, *RAD51C*, *RAD51D*, *SMAD4*, *STK11*, and *TP53*). This analysis was performed as previously described^2^ and identified a c.225dupT pathogenic variant in the *APC* gene, which was confirmed by Sanger sequencing, resulting in a premature termination codon (p.Asn76∗) (Fig. 1B; Fig. S1). *APC* is located on chromosome 5q21-q22 and consists of 15 coding exons, translating into a huge protein of 2843 amino acids. APC regulates various cellular processes.^1^ In particular, it plays a pivotal role in the Wnt signaling pathway, regulating β-catenin degradation in the cytoplasm. Loss of APC function thus results in increased nuclear levels of β-catenin and transcriptional activation of growth-promoting β-catenin target genes, which contribute to polyp formation.^1^

**Figure 1:**
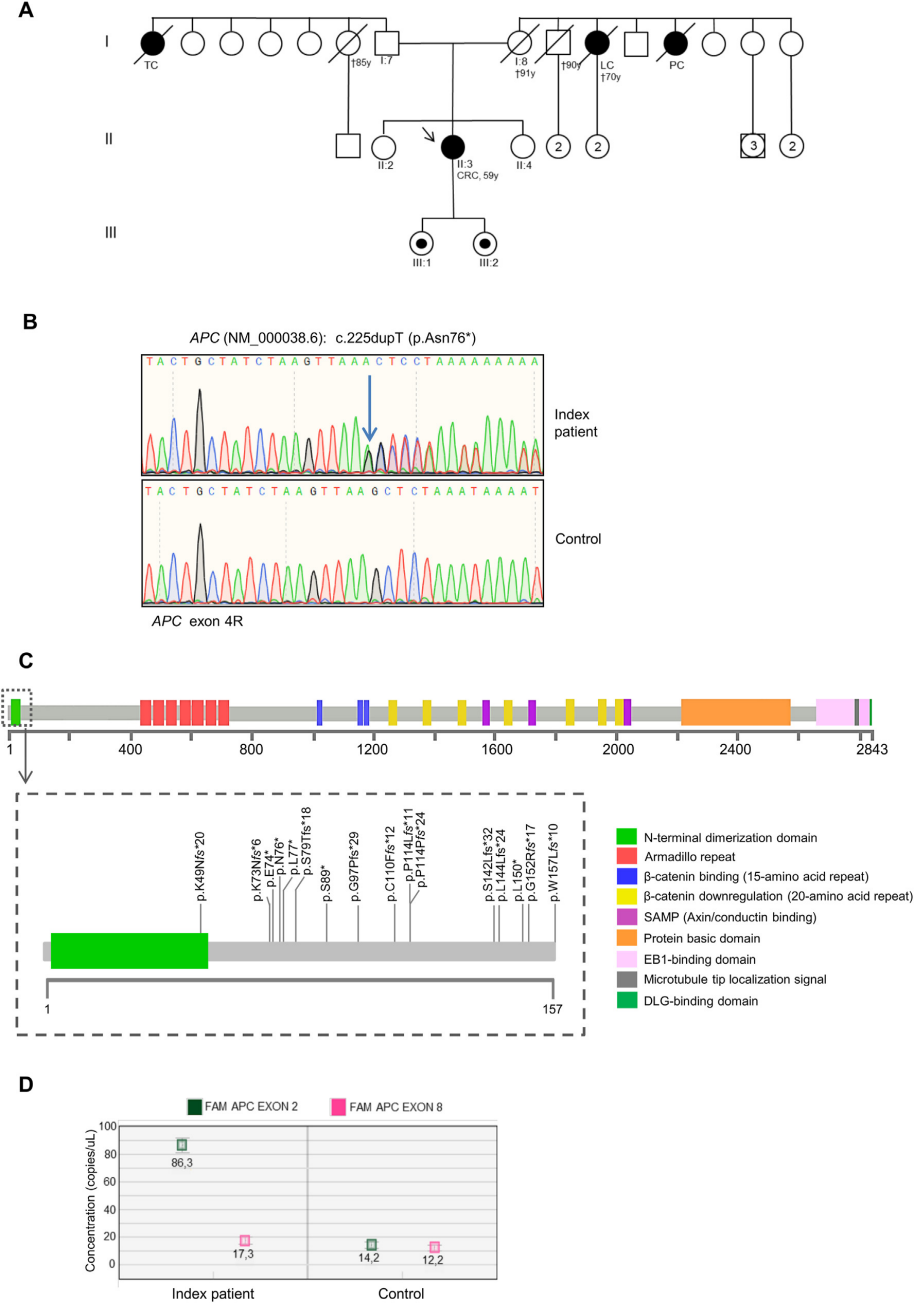
Clinical and molecular characterization of a novel germline *APC* N-terminal pathogenic variant. **(A)** Pedigree of the family involved in this study. The squares indicate men and the circles indicate women. The squares and circles with a number inside represent multiple individuals. The arrow indicates the index case. The black-filled symbols denote individuals with cancer and unfilled symbols indicate unaffected individuals. Symbols enclosing a black dot indicate unaffected individuals carrying the *APC* c.225dupT (p.Asn76∗). The slashed symbols denote dead individuals. The following information is given below each filled symbol: clinical manifestations (TC, thyroid cancer; LC, lung cancer; PC, pancreatic cancer; CRC, colorectal cancer), age of death (†), age at diagnosis (y, years). **(B)** Sequencing electropherograms of genomic DNA from the index patient, showing the *APC* c.225dupT (p.Asn76^∗^) pathogenic variant. **(C)***APC* coding region and distribution of APC N-terminal truncating variants (codons 1–157) in attenuated FAP patients identified in our literature analysis and the present study. Conserved regions and domains that interact with other proteins are shown. **(D)** Quantification results of the digital droplet polymerase chain reaction assay (copies/μL) of *APC* mRNA expression for exon 2 (FAM) and exon 8 (HEX), as processed by QuantaSoft™. mRNA extracted from an *APC* wild-type individual was used as a control. The error bars represent the maximum and minimum Poisson distribution for the 95% confidence interval generated by QuantaSoft.

*APC* c.225dupT is a novel pathogenic variant. It has not been previously described in the literature and is not listed in major disease-associated databases (HGMD Professional, ClinVar, InSight). Genetic analysis of the index patient's family members identified the *APC* c.225dupT pathogenic variant also in her daughters (Fig. 1A III:1, III:2) but not in her father and sisters (Fig. 1A I:7, II:2, II:4). The index patient's mother (Fig. 1A I:8) had already died at the time of this study; thus, her *APC* mutational status could not be assessed. However, since apparently she did not have colonic polyposis nor FAP-associated malignancies, it is reasonable to hypothesize that the germline *APC* c.225dupT is a *de novo* pathogenic variant. Likewise, previous reports estimated that about 25% of FAP patients do not have a family and/or genetic history of the syndrome, thus harboring a *de novo* *APC* pathogenic variant.

The index patient developed about 17 polypoid lesions and CRC at 59 years, exhibiting a typical attenuated FAP phenotype. Previous studies of genotype–phenotype correlation showed that the location of *APC* pathogenic variants influences FAP phenotype. In particular, classical FAP with profuse polyposis (>1000 adenomas) is associated with pathogenic variants located between codon 1250 and 1424, with an average age of 16 years (range 7–36) at diagnosis,^1^ while pathogenic variants located between codon 2052 and 2843 are associated with gastric polyposis and desmoid FAP (<50 adenomas, and desmoids tumors).^1^ Conversely, pathogenic variants located at the extreme 5′ end of the gene (codons 1–157), such as the novel pathogenic variant identified in this study, are associated with an attenuated FAP phenotype (<100 adenomas), with an average age of 50–55 years at diagnosis.^1^

To elucidate the correlation between genotype and clinical phenotype, we performed a literature analysis of *APC* N-terminal truncating variants on HGMD Professional, a comprehensive database of human inherited disease mutations. We reviewed the identified articles and collected clinical information (*i.e.*, age at diagnosis, sex, number of polypoid lesions) of patients with germline *APC* truncating variants located at the N-terminal region (codons 1–157). Patients without clinical information were excluded.

This analysis identified 35 patients with a total of only 17 unique germline *APC* truncating variants in the region encompassing codons 1–157, including the one characterized in this study. Of these, six were nonsense variants, seven were small insertions, and four were deletions (Fig. 1C and Table S1, S2). The average age at polyp diagnosis was 47.6 years (range 24–74), and most of the patients showed variable polyp numbers ranging from 10 to 100 (Table S2). Only two patients, harboring an *APC* truncating variant at codon 157 (c.469dupT), developed more than 750 polys, which could be due to the influence of other genes on phenotypic expression.^3^ In most patients (25/35), there was no evidence of malignancy at diagnosis. CRC was detected only in nine patients, for five of which the age at diagnosis was also reported. Like attenuated FAP patients, these patients showed a trend toward a later CRC onset, with an average age of 62.5 years (range 51–74) at diagnosis (Table S1). In two cases, extra-colonic manifestations (gastric cancer and hyperplastic polyposis) were the first clinical signs detected (Table S1, S2).

Overall, the results of our literature review suggested that patients harboring *APC* N-terminal pathogenic variants (codons 1–157) develop the attenuated form of FAP. The molecular bases of this correlation are unclear.^4^ Previous studies suggested that the presence of a premature termination codon may influence mRNA transcription and/or processing in addition to protein expression.^5^ We thus sought to evaluate the effect of the premature termination codon on the mRNA expression of the *APC* 225dupT mutant allele. To this end, we quantified *APC* mRNA levels upstream (exon 2) and downstream (exon 8) of the pathogenic variant in the normal tissue of our index patient by droplet digital PCR. This analysis showed that *APC* exon 2 expression levels were higher than *APC* exon 8 expression levels (Fig. 1D; Supplementary Data), suggesting a 5′-3′ transcript imbalance causing a reduction of transcript levels toward the 3′ end in the mutant allele. Indeed, it has been shown that the presence of a premature termination codon may affect DNA structure, impairing the transcription process downstream of the pathogenic variant.^5^ Moreover, due to the premature stop at codon 76, the *APC* c.225dupT allele is likely translated into a shorter protein (p.Asn76^∗^). Previous studies suggested that shorter truncated APC proteins could exert a dominant negative effect in heterozygous cells by inactivating the full-length APC wild-type protein.^4^ Although we could not test the stability of the N-terminal mutant APC protein, it has been shown that the first 170 APC amino acids are necessary for *in vitro* homodimerization, thus APC p.Ans76∗ may lead to an unstable heterodimer, thereby preserving at least in part the function of the wild-type protein. This molecular mechanism would thus provide sufficient levels of active wild-type APC, leading to the attenuated phenotype of FAP disease. The identification of this novel *APC* c.225dupT pathogenic variant and its characterization expand the knowledge of the molecular mechanisms governing *APC* genotype–phenotype correlation.

## Ethics declaration

The study was conducted according to the guidelines of the Declaration of Helsinki, and approved by the Institutional Ethics Committee of IRCCS Istituto Tumori “Giovanni Paolo II” (protocol code N 170, date of approval 31 October 2016). Informed consent was obtained from all subjects involved in the study.

## Conflict of interests

The authors declare that they have no competing interests.

## Funding

The research leading to these results has received funding from AIRC under IG 2019—ID. 23794 project—to Cristiano Simone. Furthermore, this work was funded by the research funding program “Ricerca Corrente 2021–2023″ to Cristiano Simone, “Ricerca Corrente 2022–2024″ to Vittoria Disciglio, “Ricerca Corrente 2022–2024″ to Candida Fasano, “Ricerca Corrente 2023–2025″ to Valentina Grossi, AIRC fellowship for Italy “ID. 26678–2021″ to Martina Lepore Signorile, “Starting Grant” SG-2019-12371540 to Paola Sanese from the Italian Ministry of Health, and the 2017 PRIN (Research Projects of National Relevance) n. 2017WNKSLr-LS4 from the Italian MUR to Cristiano Simone.

## References

[1] Disciglio V., Fasano C., Cariola F. (2020). Gastric polyposis and desmoid tumours as a new familial adenomatous polyposis clinical variant associated with *APC* mutation at the extreme 3'-end. J Med Genet.

[2] Disciglio V., Sanese P., Fasano C. (2022). Identification and somatic characterization of the germline *PTEN* promoter variant rs34149102 in a family with gastrointestinal and breast tumors. Genes.

[3] Walon C., Kartheuser A., Michils G. (1997). Novel germline mutations in the *APC* gene and their phenotypic spectrum in familial adenomatous polyposis kindreds. Hum Genet.

[4] Preisler L., Habib A., Shapira G. (2021). Heterozygous APC germline mutations impart predisposition to colorectal cancer. Sci Rep.

[5] García-Rodríguez R., Hiller M., Jiménez-Gracia L. (2020). Premature termination codons in the *DMD* gene cause reduced local mRNA synthesis. Proc Natl Acad Sci U S A.

