# Supplementary material for: Identification of a novel germline APC N-terminal pathogenic variant associated with attenuated familial adenomatous polyposis
Source: Genes Dis. 2023 Sep 7;11(6):101078. doi: 10.1016/j.gendis.2023.101078 (PMC11298831; doi:10.1016/j.gendis.2023.101078)
Supplement: Multimedia component 1 [file mmc1.docx]

Supplementary Data

Clinical data

The index patient (Figure 1 II:3) underwent colonoscopy due to abdominal pain, which revealed a colon neoformation in the ascending colon and many polyps extending from the rectum to the caecum. Histological examination of this colon neoformation showed a moderately differentiated adenocarcinoma, with poorly differentiated foci at the infiltration tumor front and an ulcerative formation. Moreover, this analysis showed lymphatic permeation and high-grade budding infiltrating the muscle layer of the bowel wall (pT2,N0,MX). In addition, histological analysis of the polyps showed sessile and pedunculated tubulovillous adenomas with high- and moderate-grade dysplasia. No other family members exhibited a colonic polyposis phenotype and/or FAP-associated malignancies.

RNA extraction and droplet digital PCR (ddPCR) assay

ddPCR was performed using 100 ng of RNA extracted from total blood using the miRNeasy Mini Kit according to the manufacturer’s instructions. Reactions were prepared using the One-Step RT-ddPCR Advanced Kit for Probes (Bio-Rad), according to the manufacturer’s instructions. 20 uL of each reaction mix was converted to droplets with the QX200 droplet generator (Bio-Rad). The droplets were transferred to a 96-well plate, sealed, and cycled in a C100 Thermocycler (Bio-Rad) under the following cycling protocol: 25°C for 3 min, reverse transcription at 50°C for 60 min, 95°C for 10 min followed by 40 cycles of 95°C for 30 s and 55°C for 1 min, a post-cycling step of 98°C for 10 min and inﬁnite hold at 4°C. The plate was then transferred into a QX200 Reader (Bio-Rad). The data were analyzed using Bio-Rad QX Manager 1.2 Standard Edition. Primers and probes were as follows:

- *APC* exon 8: ddPCR Gene Expression Assay, APC, Human, Homo sapiens (Bio-Rad, dHsaCPE5050550)
- *APC* exon 2: Fw primer: 5’ GTTGAGGCACTGAAGATGGAGAA; Rv primer: 5’ CTGTCCAGAAGAAGCCATAGC; probe: GGAAACTGAGGCATCTAATATGAAGGAAGT

Supplementary Tables

Supplementary Table 1. Summary of genetic and clinical features of patients carrying *APC* truncating mutations at codons 1-157 based on HGMD Professional entries.

| Total patients | 35 |
| --- | --- |
| Germline *APC* truncating mutations | 17 |
| Nonsense mutations | 6 |
| Insertions | 7 |
| Deletions | 4 |
| Average age at polyp diagnosis (range) | 47.6 (24-74) |
| CRC  Average age at CRC diagnosis (range) | 9  62.5 (51-74) |
| Extra-colonic manifestations | 4 |

Supplementary Table 2. Extended genetic and clinical features of patients carrying *APC* truncating mutation at codons 1-157 based on HGMD Professional entries.

| **Cases** | **Age of onset** | **Sex** | **Mutation** | **Predicted effect on protein** | **Number of polyps** | **Colorectal cancer (age)** | **Extracolonic manifestations (age)** | **REF** |
| --- | --- | --- | --- | --- | --- | --- | --- | --- |
| ID-9 I2 | 74 | F | c.147-150delACaa | p.Lys49Asn*fs**20 |  | Present (74) |  | Lorca et al ^1^ |
| ID-9 II1 | 60 | F |  |  | 61 | NA |  |  |
| ID-9 II1 | 35 | M |  |  | >150 | NA |  |  |
| GAL-07 | 50 | NA |  |  | 15 | Absent |  | Gomez-Fernandez et al ^2^ |
| 1 | 64 | F | c.218_219insTA | p.Lys73Asn*fs**6 | NA | Present |  | Yanus et al ^3^ |
| FAM-147 proband | 62 | M | c.220G>T | p.Glu74* | NA | NA |  | Stekrova et al ^4^ |
| FAM-147 sister | 60 | F |  |  | NA | NA |  |  |
| FAM-147 brother-1 | 54 | M |  |  | NA | NA |  |  |
| FAM-147 brother-2 | 62 | M |  |  | NA | NA |  |  |
| FAM-147 daughter-1 | 35 | F |  |  | NA | NA |  |  |
| FAM-147 daughter-2 | 38 | F |  |  | NA | NA |  |  |
| II:3 | 59 | F | c.225dupT | p.Asn76* | >17 | Present (59) |  | THIS PAPER |
| 229 | 62 | NA | c.230T>G | p.Leu77* | 100 | NA |  | Stekrova et al ^4^ |
| FAP3 | 27 | NA | c.235_236insCT | p.Ser79Thrfs*18 | 3 | Absent |  | Preisler et al ^5^ |
| ID-3 I2 | 69 | F | c.266C>G | p.Ser89* | NA | NA | Gastric cancer (69) | Lorca et al ^1^ |
| ID-3 II2 | 68 | F |  |  | >40 | Present (68) |  |  |
| ID-3 II3 | 72 | F |  |  | NA | Present (72) | Breast cancer (74) |  |
| ID-3 II5 | 56 | F |  |  | 96 | NA |  |  |
| 177 | 63 | NA | c.288_289insCC | p.Gly97Profs*29 | 100 | Absent |  | Stekrova et al ^4^ |
| FAM-59 | 51 | NA | c.329_338del10 | p.Cys110Phe*fs**12 | 15 | NA |  | de Leon et al ^6^ |
| F1195 | 49 | NA | c.340delC | p.Pro114Leu*fs**11 | 50-100 | NA |  | Fostira et al ^7^ |
| 10739201 | 42 | F | c.341dupC | p.Pro114Pro*fs**24 |  | NA |  | Chubb et al ^8^ |
| 50733 |  | NA | c.401T>A | p.Leu134* | 10-100 | Present (NA) |  | Nielsen et al ^9^ |
| FAP-2 | 48 | NA | c.423-11A>G | p.Ser142Lysfs*32 | NA | NA |  | Jarry et al ^10^ |
| 76 | 28 | NA | c.432delT | p.Leu144Leufs*24 | 100 | NA |  | Filipe et al ^11^ |
| ID-1 | 34 | NA | c.448A>T† | p.Lys150* | 40-100 | Absent | Gastric adenocarcinoma (34), gastroduodenal adenomas (34) | Tao et al ^12^ |
| ID-2 | 29 | NA | c.454_457insAGAA | p.Glu152Arg*fs**17 | 40-100 | Present (51) | Gastric hyperplastic polyps (29) | Tao et al ^12^ |
| 22.II1 | 33 | NA | c.469dupT ‡ | p.Trp157Leu*fs**10 | 750 | NA |  | Walon ^13^ |
| 22.II2 | 31 | NA |  |  | 1000 | NA |  |  |
| 22.II3 | 24 | NA |  |  | 36 | NA |  |  |
| 22.II4 | 26 | NA |  |  | 100 | NA |  |  |
| 32.I1 | 51 | NA |  |  | 100 | Present (51) |  |  |
| 32.II1 | 39 | NA |  |  | 100 | NA |  |  |
| 32.II2 | 34 | NA |  |  | 10 | NA |  |  |
| 32.II3 | 29 | NA |  |  | 100 | NA |  |  |
| NA: not available |  |  |  |  |  |  |  |  |
| † The c.448A > T (p.Lys150X) mutation is reported with a c.446A > T (p.Asp149Val) substitution on the same allele. | | | | | | |  |  |
| ‡ In the original paper, the mutation is reported as 487IinsT. | | | |  |  |  |  |  |

1. Lorca V, Rueda D, Martín-Morales L, et al. Contribution of New Adenomatous Polyposis Predisposition Genes in an Unexplained Attenuated Spanish Cohort by Multigene Panel Testing. *Sci Rep*. 2019;9(1):9814. doi:10.1038/s41598-019-46403-5

2. Gómez-Fernández N, Castellví-Bel S, Fernández-Rozadilla C, et al. Molecular analysis of the APC and MUTYH genes in Galician and Catalonian FAP families: a different spectrum of mutations? *BMC Med Genet*. 2009;10:57. doi:10.1186/1471-2350-10-57

3. Yanus GA, Akhapkina TA, Ivantsov AO, et al. Spectrum of APC and MUTYH germ-line mutations in Russian patients with colorectal malignancies. *Clin Genet*. 2018;93(5):1015-1021. doi:10.1111/cge.13228

4. Stekrova J, Sulova M, Kebrdlova V, et al. Novel APC mutations in Czech and Slovak FAP families: clinical and genetic aspects. *BMC Med Genet*. 2007;8:16. doi:10.1186/1471-2350-8-16

5. Preisler L, Habib A, Shapira G, et al. Heterozygous APC germline mutations impart predisposition to colorectal cancer. *Sci Rep*. 2021;11(1):5113. doi:10.1038/s41598-021-84564-4

6. de Leon MP, Pedroni M, Roncucci L, et al. Attenuated polyposis of the large bowel: a morphologic and molecular approach. *Fam Cancer*. 2017;16(2):211-220. doi:10.1007/s10689-016-9938-9

7. Fostira F, Thodi G, Sandaltzopoulos R, Fountzilas G, Yannoukakos D. Mutational spectrum of APC and genotype-phenotype correlations in Greek FAP patients. *BMC Cancer*. 2010;10:389. doi:10.1186/1471-2407-10-389

8. Chubb D, Broderick P, Frampton M, et al. Genetic diagnosis of high-penetrance susceptibility for colorectal cancer (CRC) is achievable for a high proportion of familial CRC by exome sequencing. *J Clin Oncol*. 2015;33(5):426-432. doi:10.1200/JCO.2014.56.5689

9. Nielsen M, Hes FJ, Nagengast FM, et al. Germline mutations in APC and MUTYH are responsible for the majority of families with attenuated familial adenomatous polyposis. *Clin Genet*. 2007;71(5):427-433. doi:10.1111/j.1399-0004.2007.00766.x

10. Jarry J, Brunet JS, Laframboise R, et al. A survey of APC mutations in Quebec. *Fam Cancer*. 2011;10(4):659-665. doi:10.1007/s10689-011-9468-4

11. Filipe B, Baltazar C, Albuquerque C, et al. APC or MUTYH mutations account for the majority of clinically well-characterized families with FAP and AFAP phenotype and patients with more than 30 adenomas. *Clin Genet*. 2009;76(3):242-255. doi:10.1111/j.1399-0004.2009.01241.x

12. Tao H, Shinmura K, Yamada H, et al. Identification of 5 novel germline APC mutations and characterization of clinical phenotypes in Japanese patients with classical and attenuated familial adenomatous polyposis. *BMC Res Notes*. 2010;3:305. doi:10.1186/1756-0500-3-305

13. Walon C, Kartheuser A, Michils G, et al. Novel germline mutations in the APC gene and their phenotypic spectrum in familial adenomatous polyposis kindreds. *Hum Genet*. 1997;100(5-6):601-605. doi:10.1007/s004390050560
