# Supplementary material for: Identification of a novel germline APC N-terminal pathogenic variant associated with attenuated familial adenomatous polyposis
Source: Genes Dis. 2023 Sep 7;11(6):101078. doi: 10.1016/j.gendis.2023.101078 (PMC11298831; doi:10.1016/j.gendis.2023.101078)
Supplement: Multimedia component 2 [file mmc2.pdf]

Supplementary Figure 1

APC (NM_000038.6): c.225dupT (p.Asn76*)

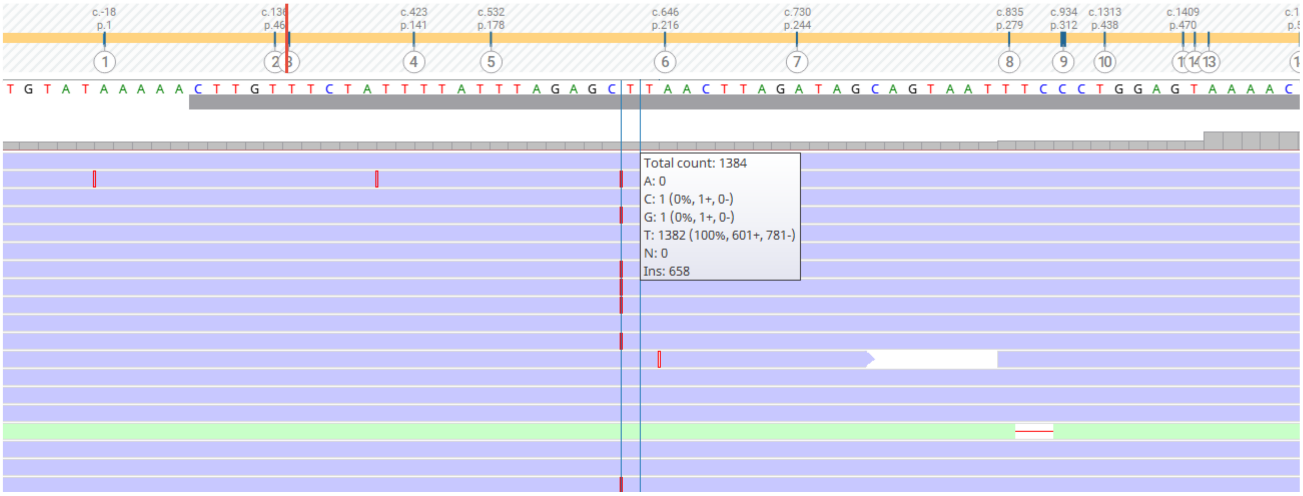

Supplementary Figure 1. Next-generation sequencing results showing the *APC* c.225dupT (p.Asn76*) pathogenic variant in genomic DNA from the index patient. Visualization of the BAM (Binary Alignment Map) file of the variant using Alamut Visual Plus software v.1.6.1 (Interactive Biosoftware, Rouen, France). A partial sequence of *APC* (NM_000038.6) is shown. The position of the c.225dupT pathogenic variant in *APC* exon 4 is highlighted by the line. The frequency and absolute number of reads are indicated for the mutant and wild-type alleles.
